# Molecular characterization of potential healthcare associated respiratory syncytial virus in three referral hospitals in Kenya, 2009-2011

**DOI:** 10.1186/2047-2994-4-S1-P20

**Published:** 2015-06-16

**Authors:** LM Mayieka, G Arunga, Z Ng'ang'a, S Khamadi, J Oundo, CL Otieno, L Ndegwa

**Affiliations:** 1 Diagnostics and Laboratory Systems Program, Kenya Medical Research Institute/Centers for Disease Control, Nairobi, Kenya; 2 Diagnostics and Laboratory Systems Program, Jomo Kenyatta University of Agriculture and Technology, Nairobi, Kenya; 3 Henry M Jackson Foundation Medical Research International, USAMRU, Tanzania, United Republic of; 4 Henry M Jackson Foundation Medical Research International, USAMRU, Kericho, Kenya; 5 International Emerging Infection Program, KEMRI/CDC, Nairobi, Kenya; 6 Infection Control Program, kenya Medical Research Institute/Centers for Disease Control, Nairobi, Kenya

## Introduction

Respiratory syncytial virus (RSV) is a major cause of community-acquired severe respiratory illness in infants, immunocompromised individuals and the elderly. Limited information exists on healthcare associated RSV infections in developing countries.

## Objectives

To describe hospital-acquired RSV infections in three Kenyan referral hospitals

## Methods

Ongoing surveillance for healthcare associated infections is conducted at three referral hospitals in Nairobi: Kenyatta National Hospital (KNH), Jaramogi Oginga Odinga Teaching and Referral Hospital (JOOTRH) and Mbagathi District Hospital (MDH). We collected nasopharyngeal and oropharyngeal samples from patients with new-onset fever (≥38°C) and either cough or sore throat, after being afebrile for at least three days in the wards. Specimen were tested for RSV using real time polymerase chain reaction (RT-PCR) and those positive with a cycle threshold value of 30 and below were further grouped as RSV A or B using the same method. The ectodomain of the attachment G glycoprotein was sequenced and phylogenetically analyzed.

## Results

Among 255 cases tested from September, 2009 to September, 2011, 37 (14.5%) were positive for RSV, including 13 (35%) subgroup A, 6 (16%) B, 1 (3%) mixed AB and 17 (46%) could not be determined. Seventeen samples were successfully sequenced out of the twenty samples on which this was attempted. Majority of our RSV A isolates belonged to NA1 genotype prototype strain and all RSV B sequences clustered with the BAIV genotype. Three RSV A and 2 RSV B sequences from patients on the same ward at KNH were 100% identical in the G ectodomain suggesting potential common source. One RSV A positive specimen from MDH and one from JOOTRH showed 100% sequence identity.

## Conclusion

Presence of identical sequences indicates potential patient to patient transmission of RSV within the hospitals. Effective and feasible infection control strategies should be enhanced in the Kenyan public hospitals.

## Disclosure of interest

None declared.

